# Integrating Refined Kano Model and QFD for Service Quality Improvement in Healthy Fast-Food Chain Restaurants

**DOI:** 10.3390/ijerph15071310

**Published:** 2018-06-22

**Authors:** Kai-Jung Chen, Tsu-Ming Yeh, Fan-Yun Pai, Der-Fa Chen

**Affiliations:** 1 Department of Industrial Education and Technology, National Changhua University of Education, Changhua 500, Taiwan; kaijung1975@yahoo.com (K.-J.C.); dfchen@cc.ncue.edu.tw (D.-F.C.); 2 Department of Industrial Engineering and Management, National Quemoy University, Kinmen 892, Taiwan; 3 Department of Business Administration, National Changhua University of Education, Changhua 500, Taiwan

**Keywords:** fast-food chain restaurants, service quality, DINESERV, refined Kano model, QFD

## Abstract

People are paying greater attention to health. To maintain a good health status and obtain food fast, customers may go to healthy fast-food chain restaurants such as Subway more often than before in China and Taiwan. Healthy fast-food chain restaurants come with a healthy spin, seeking to differentiate themselves from other fast-food restaurants. This paper combined the refined Kano model and the quality function deployment (QFD) method. The refined Kano model was used to understand how customers perceive service attributes developed based on DINESERV measurements. QFD was employed to describe the relationships among the critical service attributes and corresponding improvements as well as to identify the priority for these improvements. The analysis results revealed that providing limited offers (due to periods, seasons, and regions) should be at the top of their improvement list, followed by staff suggestions for ingredients, and a temperature display to enhance the image of fresh ingredients. Other improvement actions include providing regular launches of new flavors/products, designing new and attractive slogans, and providing restaurant apps.

## 1. Introduction

Rapid economic development and social structural changes have led to massive changes in daily life. In the past, people ate three meals at home. Today, Americans spend about half of their food dollars eating out when compared with 25% in 1955 [1], as the Taiwanese do in Asia. Fast food has become a popular alternative. In fact, it has become an integral and beloved part of life, especially for children, teenagers, or office workers on the go [2]. Fast-food restaurants or quick-service restaurants have become one of the most popular options to buy food [3,4].

The food of choice in modern life needs to be delicious, convenient, and fast [5,6]. This, combined with the popularity of prepared, fatty, high-sugar, and high-calorie food items has been a major contributor to chronic diseases such as diabetes, hypertension, and hyperlipidemia [7]. However, individual consumers are more aware and educated about their individual dietary needs, and devise dietary strategies for food choice [8,9,10]. Healthy fast-food chain restaurants such as Subway have therefore become a substitute option. These restaurants provide vegetable and fruits in sandwiches and salads. They also use natural ingredients and offer a balanced and nutritious menu in contrast with other fast-food chains. By catering to the consumers’ desire to be healthy, Subway seeks to create a new segment away from the highly competitive American fast-food market.

McDonald’s entered the Taiwanese market in 1984, followed by KFC, Wendy’s, Burger King, and MOS Burger, which came to Taiwan later. This marked the beginning for the mushrooming of Western fast-food stores and the intensification of the market competition [1,4,5]. In Taiwan, Subway is classified as fast food (as in the case of McDonald’s, KFC, and MOS Burger); however, Subway is different from the other brands. McDonald’s and KFC focus on fried foods such as hamburgers, French fries, and fried chicken. MOS Burger offers a Japanese hamburger, which also includes deep fried food. In comparison, Subway emphasizes a healthy diet. There is nothing deep fried in its menu, and all of its sandwiches are made with fresh vegetables and low-oil, low-salt meat. McDonald’s, KFC, and MOS Burger offer set menus with predetermined combinations and no options in seasoning, but Subway customers can decide what they want in their sandwiches.

There is extensive literature on the service quality of the fast-food industry, and most studies have focused on the quality elements in traditional fast-food restaurants [11,12,13,14]. Few studies have focused on fast-food restaurants that provide healthy fast food. In addition, prior research in the service quality literature has often conceptualized customer satisfaction in a one-dimensional view. This means that a higher service attribute performance leads to higher customer satisfaction levels. Recent studies have found that this relationship is nonlinear. Improvement in attribute performance will not always lead to a proportionate increase in customer satisfaction [15]. Therefore, the Kano model has been employed in the related literature to identify the service attributes that are critical for satisfaction. This model involves little mathematical computation and can be used to collect relevant information quickly. The refined Kano model improves on the traditional Kano model and thus was employed in this research. The refined Kano model was then integrated with QFD to categorize service attributes, to provide improvement actions, and to determine the ranks of these actions. Suggestions to practitioners and managers were provided to boost operating performance accordingly.

## 2. Literature Review

### 2.1. Fast-Food Chain Restaurants

The rise of the United States came with the rapid development of fast-food shops and the growing presence of American fast food in other parts of the world [2]. Block et al. [16] defined fast-food restaurants as chains with two or more of the following features: quick service, delivery services, limited or no waiting staff, and payments first. In 1984, McDonald’s opened its first store in Taiwan. Its bright, friendly, and quick service made it a hotspot for young people [5]. Later on, KFC, Wendy’s, and Burger King from the US, as well as MOS Burger from Japan, all entered the market in Taiwan. This marked the beginning of the fierce competition among fast-food chains [9], where it is essential to maintain differentiation and satisfy customers’ needs in a highly competitive market. Customer satisfaction is highly correlated with company performance [3,6]. Therefore, it is critical for companies to identify key service elements such as food quality, service quality, price and value, atmosphere, and convenience [17]. In fact, customer satisfaction influences purchase behavior [18] and creates word of mouth marketing [19].

Pollan [20] indicated that fast-food companies have industrialized the preparation of menus to meet mass demand around the world. As a result, there is public skepticism on issues regarding animal husbandry, genetically modified foods, and cooking methods (including deep frying). Meanwhile, there is rising awareness regarding light diets, well-being philosophies, and environmental friendliness. Some people have turned away from fast food. All of these factors have significantly and adversely affected the traditional fast-food industry [4,10]. As a response, some companies have started to provide healthy menus and ensure food traceability. As the world’s largest private company in fast food, Subway has always differentiated itself by offering fresh vegetables and healthy menu attributes, and so has not been impacted by the concern over unhealthy fast food.

Subway seeks to create a pleasant experience for consumers by offering friendly and professional service and allowing customers to choose healthy ingredients to their liking. The employees then prepare and season the sandwiches according to each customer’s unique wishes. In contrast with the set menus offered by its competitors, Subway provides customers with a large variety of healthier options.

### 2.2. Service Quality

Service quality is a complex concept. It is not easy for consumers to evaluate service before, during, and after consumption given the various attributes and characteristics of the rendered services [21]. Service quality is considered a key factor of customer satisfaction [22]. There is extensive literature on the relationship between service quality and customer satisfaction [23,24]. Parasuraman et al. [25] developed a conceptual model of service quality (the PZB model) which was subsequently extended into a measurement for service quality (SERVQUAL) by three scholars. They generalized service quality into 10 dimensions, and later narrowed them to five and fine-tuned the SERVQUAL measurement based on empirical studies on banks, long-distance telephone companies, credit card companies, securities brokers, and household repair/maintenance service providers [26]. The five dimensions are: tangibles, reliability, responsiveness, assurance, and empathy.

Stevens et al. [27] used the SERVQUAL measurement as the basis to develop the LOGDSERV measurement according to their empirical research findings on customers visiting fast-food restaurants, leisure restaurants, and upscale restaurants. Kim et al. [17] came up with the institutional DINESERV measurement based on the DINESERV measurement for the service quality of restaurants by referring to the five dimensions in LOGDSERV and SERVQUAL and developed a total of 29 questions. Chen and Chen [28] examined fast-food restaurants in Taiwan using the institutional DINESERV measurement developed by Kim et al. [17] to assess service quality. Service quality, food quality, price and value, atmosphere, and convenience are the key considerations for restaurants [17]. An exploratory factor analysis of the five dimensions in the institutional DINESERV measurement indicated that all five dimensions were key variables for customer satisfaction and willingness to revisit. This paper used the institutional DINESERV measurement as the framework and modified the questions according to the specifics of fast-food restaurants, the literature review, and expert opinions.

### 2.3. The Kano Model

Since the Kano model was introduced in the 1980s [29], it has become a popular model for evaluating product or service attributes and has been applied in numerous industries. The Kano model posits that key product or service attributes are related to customer satisfaction [29]. The centerpiece of this model is to analyze the nature of the product or service attributes and classify these attributes into different categories [30,31].

The Kano model improved the IPA (importance–performance analysis) limitation [32,33], one of the most popular customer-driven tools that enables companies to identify the priorities for improvement in product or service elements. In the IPA model, the relationship between element performance and overall performance is assumed to be linear and symmetric. However, some elements may have a greater effect on satisfaction than dissatisfaction, while others may show opposite patterns. The Kano model facilitates the exploration of the nonlinear and asymmetric relationship between attribute performance and customer satisfaction. It improves the IPA model and provides insights into which product or service element should be classified and prioritized. Therefore, it has become an easily used and effective tool for managers to concentrate resources on attributes with higher priorities.

The Kano model has been widely used in different fields. In the Kano model, two axes are used to classify product or service quality attributes [29,31]. The horizontal axis of the four-quadrant Kano quality model indicates the degree of implementation, and the vertical axis indicates the level of customer satisfaction. These two axes divide the quality attributes into five categories: (1) the attractive quality attributes; (2) the one-dimensional quality attribute; (3) the must-be quality attribute; (4) the indifferent quality attribute; and (5) the reverse quality attribute. The performance level of different quality elements results in varying effects on the customer’s perception of satisfaction and dissatisfaction. An increase in the level of performance of an attractive attribute enhances customer satisfaction, but a low level of performance does not specifically cause dissatisfaction. When the satisfaction of an attribute is proportional to the level of performance, it is considered a one-dimensional element. The increasing level of performance of a must-be factor does not increase satisfaction, but any decrease in this factor causes dissatisfaction. Regardless of the level of performance of an element, if an attribute results in neither satisfaction nor dissatisfaction, it is called an indifferent element [32,33].

Kondo [31] suggested that attractive quality attributes can establish differentiation and boost competitiveness, which helps to attract new customers, as well as increase market share and company profits. However, quality attributes may change over time [34]. For instance, the attractive quality may become a one-dimensional quality or a must-be quality as time goes by and consumer needs and product characteristics evolve. To determine and develop an attractive quality is crucial for companies. The Kano model was initially applied by manufacturers for quality development [29].

The Kano model can be applied to new product development or new service creation. It is also used for service quality in tourism and hospitality [3]. The Kano model has, of course, been applied to restaurant management [17] such as chain restaurants [35,36], fast-food restaurants [28,37], student restaurants, and so on, to classify service attributes and most studies have used the Kano model to improve indices of service quality. However, there are few studies that have focused on healthy restaurants. There are two potential benefits of the Kano model. First, it provides a better understanding of customer requirements by classifying them into different categories (A: Attractive Quality; O: One-dimensional Quality; M: Must-be Quality; I: Indifferent Quality; R: Reverse Quality). Second, the Kano model can be used as a prioritization tool if choices must be made. The Kano model was therefore employed in this study for service improvement in a healthy restaurant chain. For example, if the improvement of certain service attributes cannot be undertaken for financial, technical, or operational reasons, the attributes in the higher categories are given a higher priority.

More recent studies have often combined the Kano model with a different method such as importance–performance analysis (IPA) [33], regression estimating [32], the PZB model, the decision-making trial and evaluation laboratory (DEMATEL) [34], and so on, to determine the improvement priority of product and service elements in both the manufacturing and service industries. However, to determine the priority of quality elements is not its main purpose. This study aimed to find improvement strategies for attractive elements. Therefore, quality function deployment (QFD) was combined with the Kano method to decide the priority of the corresponding improvement strategies.

**Figure 1:**
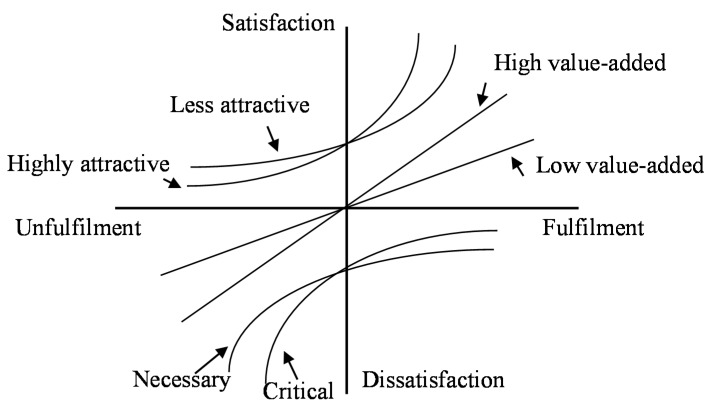
The refined Kano model.

To expand the original Kano model, Yang [38] proposed the refined Kano’s model and expanded the original four quality elements to eight attributes (see Figure 1): high attractive quality attributes, low attractive quality attributes, high value-added quality attributes, low value-added quality attributes, critical quality attributes, necessary quality attributes, potential quality attributes, and care-free quality attributes.

### 2.4. Quality Function Deployment

Akao [39] developed the theory of quality function deployment (QFD). Akao [40] indicated that QFD is a systematic approach to design quality to satisfy customers. It is about understanding the customer’s needs and the conversion of such needs into proxy attributes and product specifications. QFD is often achieved by expressing the correlation matrix between customer needs and product/service design in the form of the HOQ (house of quality) [41]. The structure of the HOQ consists of six elements, i.e., customer needs, demand assessments, engineering techniques (called service improvement in this study), correlation matrixes, engineering/technological analysis, and improvement priority.

QFD is a widely-used technique in quality management. Lin et al. [42] explored engineering techniques with the application of QFD in the design and quality improvement of restaurant services. The QFD framework covers customer needs, external service management, and after-sale service reforms. Liu et al. [43] examined restaurant services, accommodation offerings, and professional attitudes of international hotels by applying the QFD methodology and the refined Kano model with the purpose of establishing the improvement priority and key technical attributes by exploring the quality of hotel services and understanding the reason for customer dissatisfaction. Teng et al. [44] combined the Kano model and the QFD structure in the analysis of service quality in organic food stores. The QFD technique is often used in food and beverage services. Kanyan et al. [45] employed the QFD method in the examination of service quality and the exploration of customer satisfaction improvements for fast-food restaurants in Malaysia.

## 3. Research Methodology

### 3.1. Research Design

Focus group interviews were first conducted to uncover the importance service attribute for healthy fast-food chain restaurant customers. Focus group interviews are a frequently used technique for qualitative studies [46] and involve the collection of qualitative data by interviewing a group of individuals. Members in the focus group are chosen carefully to ensure that they all meet certain criteria. A moderator guides members into expressing their opinions. Focus group interviews are an efficient way to understand people’s attitudes, viewpoints, thinking, and feelings.

Focus group interviews are used in marketing, communication, psychology, education, advertising, public policy, and quality management studies [47,48,49]. Many subsequent studies of the various versions of SERQUAL have conducted empirical research using focus group interview techniques. Parasuraman et al. [25] applied the focus group interview method in the development of the 10 dimensions for service quality elements. Therefore, in this study, focus group interviews were also employed to develop service attributes in healthy fast-food chain restaurants.

Recruited participants in the focus group interviews were customers recommended by different branches of a famous healthy fast-food chain restaurant, Subway. Subway is a leading healthy fast-food chain restaurant in Taiwan and across Asia. All participants went to a healthy fast-food chain restaurants at least twice a month. In the focus groups, questions were asked in an interactive group setting where participants were free to talk with other group members. They shared their opinions about why they chose Subway rather than other kinds of fast-food restaurants, what the important service attributes were, and how they perceived these attributes. During this process, moderators took notes of the vital participants’ opinions. Important service attributes were recorded and categorized.

After obtaining the service attributes from the focus group interviews, these service attributes were compared to the DINESERV attributes [17] for fast-food restaurants and the institutional DINESERV attributes [28], and attributes not included in DINESERV or the institutional DINESERV model were then combined into these two models to identify the service attributes for healthy fast-food chain restaurants.

### 3.2. Attribute Classification

After the focus group interview, the service attributes that were important to customers but not included in the original DINESERV measurement were identified and integrated into the DINESERV attributes to develop a questionnaire based on the refined Kano model. The first part of the questionnaire contained both positive and negative questions such as “If this restaurant serves this function, how would you feel”, and “If this restaurant does not serve this function, how would you feel?”. The questionnaire used a five-point Likert scale with scores indicating strongly preferred, fairly preferred, no difference, acceptable, and strongly dislike. The second section, which collected the customers’ satisfaction of Subway, was also based on a five-point Likert scale with scores indicating very satisfied, satisfied, indifferent, acceptable, and strongly dissatisfied.

Based on the respondent’ opinions, service attributes were classified into five categories. Subway managers and customers were invited to propose and discuss improvements for attractive quality attributes and critical one-dimensional quality attributes. Finally, the QFD was employed to prioritize the improvements.

#### 3.2.1. Attribute Classifications Based on the Kano Model

The questionnaire on quality attributes based on the Kano model contained both positive and negative questions. Positive questions asked for the reaction of the customer if the service attribute was provided or fulfilled. Negative questions were concerned with the customers’ reaction if the service attribute was not provided or fulfilled.

Matzler and Hinterhuber [50] assessed the perceptions of consumers on the availability or lack of certain quality attributes and provided a systemic method to come up with five categories of quality attributes. This paper categorized quality attributes into five categories with the method proposed by Matzler and Hinterhuber [50] and labeled service quality with “A” for attractive quality, “O” for one-dimensional quality, “M” for must-be quality, “R” for reverse quality, “I” for indifferent quality, and “Q” for invalid quality (Table 1).

#### 3.2.2. Attribute Classification Based on the Refined Kano Model

The refined Kano model referred to the mean value of importance as the cutoff point for classification. If an attribute was considered an attractive quality in the Kano model, it would have a high attractive quality in the refined Kano model. If its importance value was higher than the mean value of importance of all of the attractive attributes, it was conversely considered a low attractive attribute. Table 2 shows the attribute definitions in the Kano model and the refined Kano model.

### 3.3. The Process of Quality Function Deployment

After employing the Kano model and the refined Kano model to identity the attractive quality and high value-added quality, interviews with Subway’s senior managers and customers were conducted to record proposals for improvement actions. Improvements were analyzed in the house of quality to determine implementation priorities. We constructed the house of quality according to the steps described below:

Step 1 Identify customers’ needs (WHATs): Attractive quality and high value-added quality attributes were set as the customer’ needs.
Step 2 Evaluate the importance of needs: The importance value of attractive quality and high value-added quality attributes in the refined Kano model analysis were set as the weights of these needs, respectively.
Step 3 Propose improvement actions (HOWs): Subway’s senior managers and customers discussed the improvement actions for their customers’ needs.
Step 4 Construct WHATs–HOWs correlation matrix (WHATs and HOWs): Managers from the healthy fast-food restaurant and customers discussed and came up with a correlation matrix between customer needs and improvement actions.
Step 5 Conduct improvement–action correlation analysis: Managers discussed and determined the correlations among the improvement actions.
Step 6 Prioritize improvement actions: The sum of the products of importance of each WHAT is correlated to an improvement action, and the corresponding correlation score for each improvement action was calculated. A high score indicates that the improvement action can strongly influence customer satisfaction, and therefore is a higher priority to implement.

### 3.4. Research Sampling and Data Collection

This paper examined the service quality of Subway in Taiwan by deploying the refined Kano model and the QFD method. The refined Kano questionnaire was issued to customers of American fast-food brands in Taiwan. The respondents were asked to provide their subjective assessment of food quality, service quality, price and value, atmosphere, and convenience as the five quality dimensions when they purchased sandwiches from Subway. The paper-based questionnaire was placed at the stores to reach the target customers.

## 4. Research Results

### 4.1. Analysis of Focus Group Interview Findings

Three interviews were conducted at a Subway branch in Changhua in the mornings and afternoons of 31 December 2017 and 4 February 2017. According to Subway’s senior managers, the company’s target customers are aged between 18 and 50 years. The interviewees were young and middle-aged customers who had been to Subway. Each focus group consisted of five individuals and three sessions were held, hence a total of 15 customers were interviewed, aged between 24 and 50 years, including five male and 10 female participants. The detailed information of the participants is presented in Table 3.

This study synthesized the findings from the literature review and referred to the five quality dimensions in the institutional DINESERV measurement as the framework. These five dimensions were food quality, service quality, price and value, atmosphere, and convenience. The questions on specific dimensions were modified based on the focus group interview results and Subway’s characteristics. This paper expanded the total of 18 questions in the original measurement to 20 questions, as indicated by the number of mentions and the details of the feedback from the participants (Table 4).

The three in-depth focus group interviews confirmed the list of questions for the survey. Customers who had been to Subway were invited to take part from 1 April to 31 May 2017 in 20 Subway branches. Each person was asked about their willingness to fill out questionnaires and were told that a gift, valued at around 10 dollars, would be given to them as acknowledgement after completing a questionnaire. Three hundred questionnaires were distributed in total. A total of 263 valid questionnaires were received. The response rate was 87.67%.

Most of the respondents were female (60.7%), aged 19–25 years (56.1%), students (57.6%), university educated (63.4%), earned less than NT$22,000 per month (56.9%), visited Subway fewer than five times each month (85.1%), and spent NT$101–150 per visit (57.6%). Detailed sample data are shown in Table 5.

### 4.2. The Refined Kano Model and Analysis

This study first analyzed the reliability of the 29 items and the five dimensions rated by the respondents in terms of positive and negative questions in the refined Kano model questionnaires. The Cronbach’s α values of the positive and negative questions of all five dimensions were above 0.70 individually. Thus, the results of the questionnaire were considered highly reliable.

The five dimensions of service attributes were validated via confirmatory factor analysis (CFA) using a structural equation model. The CFA results demonstrated that the positive questions of the five-dimension model fit the data well (goodness of fit index (GFI) = 0.923; adjusted goodness of fit index (AGFI) = 0.882; root mean square error of approximation (RMSEA) = 0.065). Furthermore, the negative questions of the five-dimension model had an acceptable fit to the data (GFI = 0.902; AGFI = 0.873; RMSEA = 0.078). Therefore, the service attributes effectively reflected the five dimensions.

After the validity and reliability tests, the collected data were used to classify the quality attributes into Kano attributes (see the third column in Table 6), according to Table 1. Then, the Kano attributes were further classified into refined Kano attributes according to their importance. Each kind of Kano attribute can be classified into two kinds of refined Kano attributes. Refined Kano attribute classification for each quality attribute is shown in the fourth column in Table 6.

According to Table 6, based on the refined Kano model, the high value-added attributes were: (1) overall quality of food; (2) taste of food; (3) visual appeal of the food; (4) freshness of food; (8) staff appearance; (9) attentive service; (10) staff service attitudes; (12) reliable service; (15) Chin–Chieh; (21) cleanness of facilities; (22) dining area environment; and (23) level of comfort in dining. These quality elements could ensure high levels of customer satisfaction and boost the company’s revenues. The low value-added attributes were: (19) overall value of the experience, and (27) sufficient parking space, which did not contribute much to customer satisfaction. However, a lack of these attributes could cause customer dissatisfaction. Therefore, these attributes should be offered. The high attractive quality attributes were: (5) a variety of main courses; (13) short ordering time; (14) proactive service; and (20) appropriate promotional activities. These four attributes should be prioritized. It is worth noting that the satisfaction levels and importance degrees assigned to (20) appropriate promotional activities were lower than the sample means. Therefore, Subway was advised to make improvements in this regard to enhance the level of customer satisfaction and the presence of its brand to consumers.

The only potential quality attribute identified was (11) staff professional knowledge, which has the potential to attract customers. If financially possible, the company concerned should enhance the implementation of this item and boost it as an attractive quality to consumers. The care-free quality attributes were: (6) a variety of side dishes to choose from; (7) a variety of sources to choose from; (17) good value for the money; (18) appropriate portion size; (25) sufficient tables and seats; (26) convenient location; (27) short walking distance; (16) quick takeaways with prepared meals; and (29) offering of drive-through services. If cost is an issue, it is possible to reduce or simply not offer these services.

Among the one-dimensional quality attributes, a total of 12 attributes were classified as being high value-added due to the high levels of emphasis by consumers. Understandably, Subway should enhance the offering of high value-added services (in contrast with low value-added services). Given the strong emphasis by consumers, these services are more likely to ensure customer satisfaction and boost the firm’s performance. In fact, these are also the reasons why Subway remains popular today. In addition, the company should seek to maintain their competitive advantage. On the other hand, low value-added service attributes, albeit less important, are still one-dimensional qualities, and Subway should stay diligent in the rendering of these service elements.

The more important indifferent attributes were classified as potential qualities. This study identified one potential quality, i.e., professional knowledge of service personnel. Therefore, Subway should put more effort into enhancing the professional knowledge of the service staff so that this service attribute can be converted into an attractive one. Less important attributes were defined as indifferent qualities. A total of nine indifferent attributes were identified by this paper. This suggests that consumers do not care about Subway’s prior efforts and investment in these areas, and that the continued offering of these services will not satisfy customers. Therefore, these services should be reduced or stopped.

Highly attractive quality attributes were measured by the level of importance. This paper identified four such attributes. If Subway wants to surpass competitors by developing new clientele, these four attributes should be prioritized in order to create differentiation and enhance competitiveness. The less important attractive service attribute was the promotional activities. The step-up in the offering of flexible promotion schemes could enhance the level of customer satisfaction and the importance of Subway amongst its consumers.

The less important, must-be quality was a reasonable price. Subway should be cautious with pricing so that customers do not feel that the price is too high or too low for its goods and services.

### 4.3. QFD Analysis and Findings

**Figure 2:**
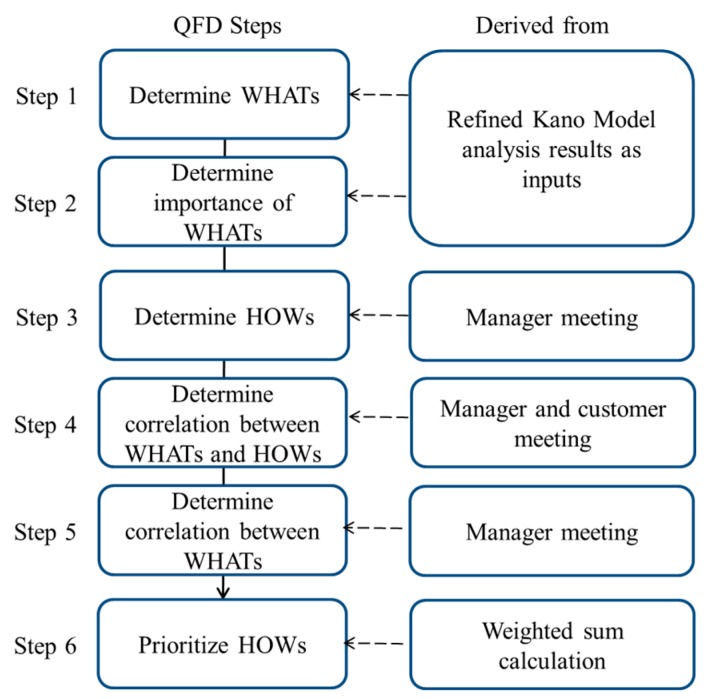
Quality function deployment (QFD) analysis process.

Five Subway managers in different areas in Taiwan were invited to give their opinions to determine the improvement actions (HOWs) required to meet the needs of the customer (WHATs). The purpose was to construct a correlation matrix between the customer needs (WHATs) and improvement actions (HOWs). The assessed weights for customer needs were the inputs for the correlation matrix to derive the weights for the improvement actions in order to prioritize the desired improvement actions in the QFD method. The process for the QFD analysis is demonstrated in Figure 2.

Customer needs (WHATs) were identified by screening the four attractive quality attributes and 12 high value-added attributes in the Kano model and the refined Kano model. The consensus from Subway’s senior managers was that the four attractive attributes should be customer needs. However, based on experience and professional knowhow, five managers then condensed these 12 high value-added attributes into four: overall food quality, taste of the food, freshness of food, and service attitude.

A demand assessment was conducted by assigning the importance of the eight quality attributes for customer needs. The degrees of importance were as follows: a variety of main courses to choose from (4.32); short ordering time (4.42); proactive services (4.41); appropriate promotional activities (4.10); overall food quality (4.36); taste of the food (4.38); freshness of food (4.50); and service attitudes (4.51), as shown in Table 6. The derived weights for the importance assigned to the customer needs attributes were the inputs for the demand assessment.

Improvement actions (HOWs) were defined by the Subway managers based on their experience and professional knowhow according to the eight quality attributes for customer needs. Four managers from four branches in four different areas were invited to provide their suggestions for improvement actions. They all had at least five years of experience in healthy fast-food restaurants or more. For instance, the management believed that a temperature display for the chilled fresh ingredients could inspire confidence in consumers regarding the freshness of Subway’s food, which could improve their perceptions of food freshness and food quality in general. Meanwhile, one of Subway’s key ingredients is bread. If consumers know when the fresh bread is taken out of the oven, they can have a better feel for the freshness of Subway’s bread. This will enhance their perceptions of the food quality in general. Hence, management came up with the idea of displaying the temperature of the chilled section and the time when fresh bread was ready from the oven. Furthermore, Subway’s senior managers stated that the development of Subway apps could accelerate the turnaround because customers would be able to order online and avoid queuing. Apps could also serve as a campaign vehicle for Subway to increase the frequency of promotional offerings.

Attractive slogans articulate the hallmark of their service attitude. Subway’s managers stated that if service personnel spoke the slogans in a loud and clear manner, consumers will perceive their service attitude positively, which will also enhance Subway’s brand image. Other suggestions include ingredient mix-and-match as a complementary service to quick ordering, proactive service, and good attitude. Management believed that good advice on ingredient combinations for new or indecisive customers may speed up the turnover and accelerate the ordering workflow. Regular launches of new flavors/products, limited supplies (due to time periods, seasons, and regions), and freshly squeezed juices are services that could ensure a variety of main courses to choose from and improve the attractiveness of Subway menus. Senior management also believed that these three services would provide additional options to consumers. The launch of new flavors or offering products with limited supplies could continue to excite customers about going to Subway as well as make Subway’s food more attractive.

**Figure 3:**
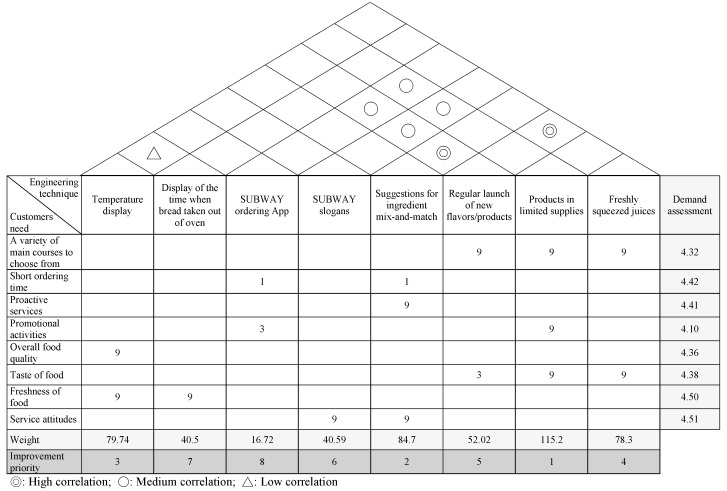
The house of quality for Subway.

After listing the eight service improvement actions, Subway’s senior managers determined the correlations between each pair of customer needs (WHATs). They then held a discussion with four Subway customers regarding the correlation level between customer needs (WHATs) and improvements (HOWs) to complete the correlation matrix by assigning high, medium, and low correlations based on the consensus views. This was followed by the multiplication of the correlation levels and the weights of customer needs for each improvement action. The sum of the products, a weighted sum for each improvement action, was the basis for improvement action prioritization (Figure 3). From Figure 3, the weighted sum of the limited offer was 115.2 and is therefore the first priority for a company to implement.

The deployment and analysis of the house of quality was used to derive the improvement priority of the eight improvements and is shown in Table 6.

According to the data shown in Table 7, limited offers (due to time periods, seasons, and regions) should be at the top of the priority list, followed by suggestions for ingredient mix-and-match. The third service on the priority list was the temperature display for the chilled section on a real-time basis. This will enhance Subway’s image as a provider of fresh ingredients. Other items on the priority list in order of importance were the offering of freshly squeezed juices, regular launches of new flavors/products, new slogans, time display for when bread is taken out of the oven, and restaurant apps to provide products, nutrition, and promotion information.

## 5. Conclusions

In this study, the refined Kano model and QFD were combined to determine the attributes of quality elements in fast-food chain restaurants and find the improvement strategies by QFD.

From the analysis results of the refined Kano model, a total of 14 quality elements were identified as a one-dimensional attribute. Furthermore, 12 of these quality attributes were categorized as high value-added. This means that Subway should put extra effort into these one-dimensional quality attributes.

The attractive attribute is the strategic starting point of differentiating from peers as well as the source of key competitiveness. A lack of attractive attributes will not upset customers, but their perceived satisfaction will improve when they are made available. This paper identified four attractive attributes, and health fast-food restaurants should consider offering these services. Attractive attributes can be further divided into high attractive ones and low attractive ones, according to their level of importance. These are opportunities for fast-food restaurants to boost customer satisfaction.

Four attractive and four selected one-dimensional attributes were chosen in the house of quality and eight improvements were derived according to these eight service attributes. Although it is not easy to improve the variety in fast-food restaurants, management still tries their best to increase the variety of main courses. For example, Subway may explore alternatives to bread such as potatoes or sweet potatoes as a staple to go with meat and vegetables. It may also be possible to provide additional meat options. Furthermore, restaurants can explore the use of ordering machines or app ordering in addition to the existing manual ordering process and the requirement of queuing in store. Regular customers may simply use a machine or tap on an app to order. Non-regular customers may be given advice regarding ingredient mix-and-matching to shorten the waiting time for those unfamiliar with Subway products or procedures. This will shorten the queue and speed up the ordering process.

To ensure proactive services, healthy fast-food restaurants should foster a relevant corporate culture with appropriate personnel training. Employees should be encouraged to inquire about the needs and feelings of customers to be able to immediately make improvements if necessary. They can also offer assistance by providing professional advice for customers to create their own delicious sandwiches. Subway may also publish secret recipes and demonstrate its knowhow to impress and excite customers.

Services, food, and beverages can be easily copied by competitors. Therefore, a restaurant should make sure that it is able to create unique value by extending its image as a healthy restaurant using fresh ingredients. Décor, publicity, and strategic alliances with other industries should all be integrated into its services. The rising awareness in environmental protection has helped to augment Subway’s presence with existing customers and made it easier to attract different customer segments (e.g., young adults who pay attention to diet). This can create great differentiation between the competitors and change the stereotype that traditional fast food is junk food by making a healthy fast-food restaurant a company that can keep or improve their customers’ health status.

In addition to the suggestions for health fast-food chain restaurants, other kinds of restaurants can also adopt the proposed combined method to find the attributes of service elements and development improvements for the service. They can understand their customers more and figure out improvements to satisfy them. Managers can therefore find a way to differentiate their restaurants from their competitors, develop new customers, and sustain old ones.

This study simply focused on service attributes and the question of how to improve the performance of these service attributes. For a restaurant, the cost of maintaining regular customers is much lower than the cost of developing new customers. Therefore, future study will explore how a restaurant keeps customers or turns them into loyal ones to increase customer lifetime value.

## Figures and Tables

**Table 1:** Quality attribute classification in the Kano model.

Customer Preference	Dysfunctional Form of the Questions (Negative Questions)				
	Like	Must-be	Neutral	Live with	Dislike
Functional Form of the Questions (Positive Questions)					
Like	Q	A	A	A	O
Must-be	R	I	I	I	M
Neutral	R	I	I	I	M
Live with	R	I	I	I	M
Dislike	R	R	R	R	Q

**Table 2:** Attribute definitions in the Kano model vs. the refined Kano model.

Kano Model	Refined Kano Model	
Quality Attribute	High Important Attributes	Low Important Attribute
Attractive quality	High attractive quality	Low attractive quality
One-dimensional quality	High value-added quality	Low value-added quality
Must-be quality	Critical quality	Necessary quality
Indifferent quality	Potential quality	Care-free quality

**Table 3:** Participant information in focus group (*n* = 15).

Item	Breakdown	No.	Item	Breakdown	No.
Gender	Male	5	Visits per month	Less than 1	3
	Female	10		Less than 2	4
Age	25 years old or below	4		Less than 4	5
	26–35 years old	3		More than 5	3
	36–45 years old	5	Major transpiration (how interviewees visit Subway)	Walking	5
	Older than 46 years	3		Motorcycle	5
Occupation	Military, governmental employees, and teachers	3		Motors	5
	Manufacturing industry	3			
	Business	5			
	Healthcare	1			
	Student	3			

**Table 4:** Focus group interview findings.

Dimension	Service Attributes in DINESERV	Service Attributes from Focus Group Interviews	No. of Mentions	No. of Customer Mentions	%
Food quality	1. Overall quality of the food	1. Overall quality of the food	20	15	6.68%
	2. Taste of the food	2. Taste of the food	9	7	3.01%
	3. Visual appeal of the food	3. Visual appeal of the food	22	14	7.35%
	4. Freshness of the food	4. Freshness of the food	22	15	7.35%
		5. A variety of main courses (new) 6. A variety of side dishes (new) 7. A variety of sauces (new)	9	5	3.01%
Service quality	5. Staff appearance	8. Staff appearance	17	15	5.68%
	6. Attentive services	9. Attentive services	15	14	5.01%
	7. Staff’s service attitudes	10. Staff’s service attitudes	13	13	4.34%
	8. Staff’s professional knowledge about the food	11. Staff’s professional knowledge about the food	13	13	4.34%
	9. Reliable services	12. Reliable services	25	15	8.36%
		13. Short ordering time (new) 14. Proactive services (new) 15. Chin-Chieh (new) 16. Quick takeaways with prepared meals (new)	6	5	2.00%
Price and value	10. Good value for money	17. Good value for money	18	14	6.02%
	11. Appropriate portion size	18. Appropriate portion size	13	11	4.34%
	12. Reasonable prices	19. Reasonable prices	20	13	6.68%
	13. Overall value of the dining experience	20. Overall value of the dining experience	16	14	5.35%
		21. Appropriate promotional activities (new)			
Atmosphere	14. Cleanness of facilities	22. Cleanness of facilities	16	13	5.35%
	15. Dining area environment	23. Dining area environment	19	13	6.35%
	16. Level of comfort in the dining	24. Level of comfort in the dining	7	6	2.34%
		25. Sufficient tables and seats (new)			
Convenience	17. Convenient location	26. Convenient location	7	7	2.34%
	18. Short walking distance	27. Short walking distance	12	8	4.01%
		28. Sufficient parking space (new)			
		29. Offering of drive-through services (new)			
TOTAL			299		100%

**Table 5:** Descriptive statistical data for the Kano model respondents.

Item	Breakdown	No.	%	Item	Breakdown	No.	%
Gender	Male	105	39.9	Spending per visit	<NT$100	64	24.4
	Female	158	60.1		NT$101–150	151	57.6
Visits per month	5 times or less	223	85.1		NT$151–200	39	14.9
	6–10 times	27	10.3		NT$201–300	7	2.3
	11–15 times	7	2.7		>NT$301	2	0.8
	16 times or more	6	1.9	Educational level	High school or below	48	18.2
Age	25 years old or below	167	63.7		Bachelor’s degree	166	63.1
	26–35 years old	61	23.3		Graduate school	49	18.7
	36–45 years old	18	6.5	Occupation	Military, governmental employees, and teachers	16	6.1
	46 years old or above	17	6.5		Labor	9	3.4
Monthly income	<NT$30,000	149	56.8		Business	49	18.3
	NT$30,000–60,000	92	35.1		Healthcare	15	5.7
	NT$60,000–90,000	17	6.1		Student	151	57.6
	>NT$90,000	5	2.0		Others	23	8.8

**Table 6:** Quality attribute classification.

Dimension	Modified Question	Kano Attribute	Refined Kano Attribute	Importance
Food quality	1. Overall quality of the food	One-dimensional	High value-added	4.36
	2. Taste of the food	One-dimensional	High value-added	4.42
	3. Visual appeal of the food	One-dimensional	High value-added	4.38
	4. Freshness of the food	One-dimensional	High value-added	4.50
	5. A variety of main courses	Attractive	Highly attractive	4.32
	6. A variety of side dishes	Indifferent	Care-free	3.89
	7. A variety of sauces	Indifferent	Care-free	4.19
Service quality	8. Staff appearance	One-dimensional	High value-added	4.48
	9. Attentive services	One-dimensional	High value-added	4.47
	10. Staff’s service attitudes	One-dimensional	High value-added	4.51
	11. Staff’s professional knowledge about the food	Indifferent	Potential quality	4.33
	12. Reliable services	One-dimensional	High value-added	4.47
	13. Short ordering time	Attractive	Highly attractive	4.42
	14. Proactive services	Attractive	Highly attractive	4.41
	15. Chin–Chieh	One-dimensional	High value-added	4.50
	16. Quick takeaways with prepared meals	Indifferent	Care-free	3.84
Price and value	17. Good value for money	Indifferent	Care-free	4.12
	18. Appropriate portion size	Indifferent	Care-free	3.96
	19. Reasonable prices	Must-be	Necessary	4.01
	20. Overall value of the dining experience	One-dimensional	Low value-added	4.33
	21. Appropriate promotional activities	Attractive	Highly attractive	4.10
Atmosphere	22. Cleanness of facilities	One-dimensional	High value-added	4.50
	23. Dining area environment	One-dimensional	High value-added	4.39
	24. Level of comfort in the dining	One-dimensional	High value-added	4.36
	25. Sufficient tables and seats	Indifferent	Care-free	4.02
Convenience	26. Convenient location	Indifferent	Care-free	4.24
	27. Short walking distance	Indifferent	Care-free	4.13
	28. Sufficient parking space	One-dimensional	Low value-added	4.17
	29. Offering of drive-through services	Indifferent	Care-free	3.84

**Table 7:** Improvement prioritization in house of quality.

Improvement Actions (HOWs)	Weighted Sum	Priority
Limited offers	115.2	1
Suggestions for ingredients mix-and-match	84.7	2
Temperature display	79.74	3
Freshly squeezed juices	78.3	4
Regular launch of new flavors/products	52.02	5
New and attractive slogans	40.59	6
Display of the time when bread taken out of oven	40.5	7
Restaurant app	16.72	8

## References

[1] Gittelsohn J., Seung H.L.K., Batorsky B. (2013). Community-Based Interventions in Prepared-Food Sources: A Systematic Review. Prev. Chronic Dis..

[2] Tsai C.C., Ou I.C., Wu K.L. (2011). A Study of the Relationship among Fast-Food Restaurants Customers’ Experience Quality, Perceived Value, and Satisfaction—The Case of McDonald & MOS Burger. Int. J. LISREL.

[3] Chang P.T., Sung Y.C. (2012). The Study of Relationship between Customers’ Purchasing Intention and Service Quality in Fast Food Restaurant. J. Taiwan Hosp. Tour..

[4] Huang C.I., Sun C.L. (2013). The Study of McDonald's Outlet Innovation Strategy: The Cases of Hsinchu and Miaoli Outlet. Yu Da Acad. J..

[5] Hai H.L., Wang K.M., Huang S.K., Lin F.Y., Chou W.J., Lin C.W. (2014). Using AHP to Assess Critical Success Factors of MacDonalds’ Marketing Management: A Case Study at Qishan District Kaohsiung. Manag. Inf. Comput..

[6] Hsiao Y.D., Chang J.C., Wu S.T., Tang H.L., Chung K.Y. (2016). The Study of College Students MacDonald Satisfaction: Example for Chungyu Institute of Technology. J. Glob. Manag. Econ..

[7] Norgren L., Hiatt W.R., Dormandy J.A., Nehler M.R., Harris K.A., Fowkes F.G.R. (2007). Inter-society consensus for the management of peripheral arterial disease. Eur. J. Vasc. Endovasc. Surg..

[8] Chu P.C., Su M.H., Yang H.J., Kuo P.H. (2015). The associations among unhealthy eatinghabits, badeating experiences and depression in Taiwanese youths. Taiwan J. Public Health.

[9] Chen M.L. (2015). The Level of Understanding towards Health Awareness, Lifestyle and Dietary Behavior and the Impact Factors Thereof—A Case Study of a Seashore Town in Central Taiwan. J. Health Sci..

[10] Chen H.H. (2011). A Study of Taiwan Fast Food Industry Strategic. J. Sport Leis. Hosp. Res..

[11] Oyewole P. (1999). Multi-attribute dimensions of service quality in the fast food restaurant industry. J. Restaur. Foodserv. Mark..

[12] Qin H., Prybutok V.R., Zhao Q.L. (2010). Perceived service quality in fast-food restaurants: Empirical evidence from China. Int. J. Qual. Reliab. Manag..

[13] Mondurailingam M., Jeyaseelan V., Subramani A.K. (2015). Comparative Study on Customer Satisfaction towards KFC and McDonalds, Chennai. ZENITH Int. J. Multidiscip. Res..

[14] Izogo E.E., Ogba I.E. (2015). Service quality, customer satisfaction and loyalty in automobile repair services sector. Int. J. Qual. Reliab. Manag..

[15] Matzler K., Bailom F., Hinterhuber H.H., Renzl B., Pichler J. (2004). The asymmetric relationship between attribute-level performance and overall customer satisfaction: A reconsideration of the importance–performance analysis. Ind. Mark. Manag..

[16] Block J.P., Scribner R.A., DeSalvo K.B. (2004). Fast Food, Race/Ethnicity, and Income: A Geographic Analysis. Am. J. Prev. Med..

[17] Kim W.G., Ng C.Y.N., Kim Y.S. (2009). Influence of institutional DINESERV on customer satisfaction, return intention, and word-of-mouth. Int. J. Hosp. Manag..

[18] Intter C.D., Larcker D.F. (1998). Are non-financial measures leading indicators of financial performance? An analysis of customer satisfaction. J. Account. Res..

[19] Athanassopoulos A.D. (2000). Customer satisfaction cures to support market segmentation and explain switching behavior. J. Bus. Res..

[20] Pollan M. (2007). The Omnivore’s Dilemma: A Natural History of Four Meals.

[21] Bougoure U.S., Neun M.K. (2010). Service Quality in the Malaysian Fast Food Industry: An Examination Using DINESERV. Serv. Mark. Q..

[22] Gotlieb J.B., Grewal D., Brown S.W. (1994). Consumer Satisfaction and Perceived Quality: Complementary or Divergent Constructs?. J. Appl. Psychol..

[23] Storbacka K., Lehtinen J.R. (2001). Customer Relationship Ship Management: Creating Competitive Advantage through Win-Win Relationship Strategies.

[24] Parsa H.G., Gregory A., Self J.T., Dutta K. (2012). Consumer behaviour in restaurants: Assessing importance restaurant attributes in consumer patronage willingness pay. J. Serv. Res..

[25] Parasuraman A., Zeithaml V.A., Berry L.L. (1985). A Conceptual Model of Service Quality and Its Implications for Future Research. J. Mark..

[26] Parasuraman A., Zeithaml V.A., Berry L.L. (1988). SERVQUAL: A Multiple-Item Scale for Measuring Consumer Perceptions of Service Quality. J. Retail..

[27] Stevens P., Knutson B., Patton M. (1995). DINESERV: A tool for measuring service quality in restaurants. Cornell Hotel Restaur. Adm. Q..

[28] Chen H.T., Chen B.T. (2015). Integrating Kano Model and SIPA Grid to Identify Key Service Attributes of Fast Food Restaurants. J. Qual. Assur. Hosp. Tour..

[29] Kano N., Seraku N., Takahashi F., Tsuji S. (1984). Attractive Quality and Must-be Quality. J. Jpn. Soc. Qual. Control.

[30] Luor T., Lu H., Chien K., Wu T. (2015). Contribution to quality research: A literature review of Kano’s Model from 1998 to 2012. Total Qual. Manag. Bus. Excell..

[31] Kondo Y. (2000). Attractive quality: Its importance and the points of remark. Total Qual. Manag. Bus. Excell..

[32] Shen X.X., Tan K.C., Xie M. (2000). An Integrated Approach to Innovation Product Development Using Kano’s Model and QFD. Eur. J. Innov. Manag..

[33] Wu H.H., Tang Y.T., Shyu J.W. (2010). An Integrated Approach of Kano’s model and Importance-Performance Analysis in Identifying Key Success Factors. Afr. J. Bus. Manag..

[34] Yeh T.M. (2010). Determining medical service improvement priority by integrating the refined Kano model, Quality function deployment and Fuzzy integrals. Afr. J. Bus. Manag..

[35] Chen L.F. (2014). A novel framework for customer-driven service strategies: A case study of a restaurant chain. Tour. Manag..

[36] Pai F.Y., Yeh T.M., Tang C.Y. (2018). Classifying restaurant service quality attributes by using Kano model and IPA approach. Total Qual. Manag. Bus. Excell..

[37] Baran Z., Yıldız M. (2015). Quality Function Deployment and Application on a Fast Food Restaurant. Int. J. Bus. Soc. Sci..

[38] Yang C.C. (2005). The refined Kano’s model and its application. Total Qual. Manag. Bus. Excell..

[39] Akao Y. (1972). New product development and quality assurance deployment system. Standardisation Qual. Control.

[40] Akao Y. (1990). Quality Function Deployment: Integrating Customer Requirements into Product Design.

[41] Hauser J., Clausing D. (1988). The House of Quality. Harvard Bus. Rev..

[42] Lin Y.H., Yu D.Y., Huang C.Y. (2005). Employing a Hypothetical Model to Investigate the Application of Quality Function Deployment (QFD) in a Restaurant’s Service Design and Quality Improvement. J. Hosp. Home Econ..

[43] Liu M.S., Wu S.D., Lu M.C., Huang Y.C., Hsu W.C., YU W.J., Tseng C.P. (2012). In application with Integration of QFD and Refined Kano Model Analysis to explore the service Quality of hotel—A case study of an anonymous hotel Tainan city. J. Far East Univ..

[44] Teng T.I., Chen Y.C., Lee Y.C., Peng K.C. (2015). Using Kano’s Model and Quality Function Deployment to Investigate the Service Quality of Organic Specialty Store in Pingtung County. J. Agric. Assoc. Taiwan.

[45] Kanyana A., Ngana L., Voonc B.H. (2016). Improving the Service Operations of Fast-food Restaurants. Procedia Soc. Behav. Sci..

[46] Webb C., Kevern J. (2001). Focus groups as a research method: A critique of some aspects of their use in nursing research. J. Adv. Nurs..

[47] Stewart D.W., Shamdassani P.N. (1990). Focus Groups: Theory and Practice.

[48] Loriz L.M., Foster P.H. (2001). Focus groups: Powerful adjuncts for program evaluation. Nurs. Forum.

[49] Wilhelmsson S., Foldevi M. (2003). Exploring views on Swedish district nurses’ prescribing-a focus group study in primary health care. J. Clin. Nurs..

[50] Matzler K., Hinterhuber H.H. (1998). How to Make Product Development Projects More Successful by Integrating Kano’s Model of Customer Satisfaction into Quality Function Deployment. Technovation.

